# Differences in antibiotic and antiviral use in people with confirmed influenza: a retrospective comparison of rapid influenza PCR and multiplex respiratory virus PCR tests

**DOI:** 10.1186/s12879-021-06030-w

**Published:** 2021-04-07

**Authors:** Victor Au Yeung, Kiran Thapa, William Rawlinson, Andrew Georgiou, Jeffrey J. Post, Kristen Overton

**Affiliations:** 1 Department of Infectious Diseases, Prince of Wales Hospital, Randwick, Sydney, NSW Australia; 2 Serology and Virology Division (SAViD), Prince of Wales Hospital, Barker Street, Randwick, NSW Australia; 3 School of Medical Sciences, School of Biotechnology and Biomolecular Sciences, School of Women’s and Children’s Health, University of New South Wales, Sydney, NSW Australia; 4 Australian Institute of Health Innovation, Faculty of Medicine, Health and Human Sciences, Macquarie University, Sydney, NSW Australia; 5 Prince of Wales Clinical School, University of New South Wales, Randwick, NSW Australia

**Keywords:** Influenza, Rapid polymerase chain reaction, Oseltamivir, Antimicrobial stewardship

## Abstract

**Background:**

Influenza is a highly contagious respiratory virus with clinical impacts on patient morbidity, mortality and hospital bed management. The effect of rapid nucleic acid testing (RPCR) in comparison to standard multiplex PCR (MPCR) diagnosis in treatment decisions is unclear. This study aimed to determine whether RPCR influenza testing in comparison to standard MPCR testing was associated with differences in antibiotic and antiviral (oseltamivir) utilisation and hospital length of stay in emergency department and inpatient hospital settings.

**Methods:**

A retrospective cohort study of positive influenza RPCR and MPCR patients was performed utilising data from the 2017 influenza season. Medical records of correlating patient presentations were reviewed for data collection. An analysis of RPCR versus MPCR patient outcomes was performed examining test turnaround time, antibiotic initiation, oseltamivir initiation and hospital length of stay for both emergency department and inpatient hospital stay. Subgroup analysis was performed to assess oseltamivir use in high risk populations for influenza complications. Statistical significance was assessed using Mann-Whitney test for numerical data and Chi-squared test for categorical data. Odds ratio with 95% confidence intervals were calculated where appropriate.

**Results:**

Overall, 122 RPCR and 362 MPCR positive influenza patients were included in this study. Commencement of antibiotics was less frequent in the RPCR than MPCR cohorts (51% vs 67%; *p* < 0.01, OR 0.52; 95% CI 0.34–0.79). People at high risk of complications from influenza who were tested with the RPCR were more likely to be treated with oseltamivir compared to those tested with the MPCR (76% vs 63%; *p* = 0.03, OR 1.81; 95% CI 1.07–3.08). Hospital length of stay was not impacted when either test was used in the emergency department and inpatient settings.

**Conclusions:**

These findings suggest utilisation of RPCR testing in influenza management can improve antibiotic stewardship through reduction in antibiotic use and improvement in oseltamivir initiation in those at higher risk of complications. Further research is required to determine other factors that may have influenced hospital length of stay and a cost-benefit analysis should be undertaken to determine the financial impact of the RPCR test.

## Introduction

Influenza is a highly contagious respiratory virus with significant impacts on morbidity and mortality in the elderly, immunocompromised and those with chronic health conditions such as chronic obstructive pulmonary disease [1]. Early detection can lead to treatment with antiviral therapy in high risk individuals [2] and could impact hospital bed management and resource allocation such as isolation rooms, subsequent bed flow and personal protective equipment. The 2017 Australian influenza season recorded some of the highest levels of influenza activity since the 2009 pandemic with 2.3 times more influenza related admissions compared to the average number of admissions from 2012 to 2016 [3]. Globally, it has been estimated that approximately 9.5 million influenza associated hospitalisations occur annually, with approximately 145,000 deaths [4]. The highest mortality rate is in those greater than 70 years old at 16.4 deaths per 100,000 [4]. Though the majority of influenza illnesses may be self-limiting, it has been estimated to be associated with 4–6 working days lost per infection case [5].

As influenza may present with a lower respiratory tract infection it is frequently treated as bacterial pneumonia. A diagnosis of influenza may reduce unnecessary antibacterial prescriptions and increase the appropriate use of antivirals for influenza. Oseltamivir treatment for influenza can reduce the duration of illness by approximately 1 day and has benefit in people with severe influenza [6, 7]. Unnecessary antibacterial therapy is associated with risks of *Clostridioides difficile* infection, adverse effects such as acute kidney injury and the development of antimicrobial resistance [8–10].

Earlier rapid influenza tests that were antigen based demonstrated poor sensitivity in detecting influenza and randomised controlled trials (RCT) failed to demonstrate improvement in clinical outcomes [11, 12]. Rapid PCR (RPCR) testing has greater specificity and sensitivity than rapid antigen testing [13]. Data on the effects of RPCR testing on antiviral and antibacterial therapy prescribing are limited. Most of the available data on the effects of rapid influenza testing on outcomes for people presenting to Emergency Departments include non-comparative cohort studies [14–16] or use historical controls [17–23]. These studies have generally compared a RPCR test to a multiplex PCR (MPCR) test with longer turnaround times. They have suggested the potential for reductions in antibacterial therapy prescription, increased antiviral prescription or effects on admission rates or infection control procedures. The lack of controls or the use of before and after test implementation design may affect the conclusions of these studies as there is significant variation in circulating seasonal influenza viruses and magnitude of cases which may affect clinician decision making.

We aimed to determine whether RPCR influenza testing was associated with differences in antibacterial and antiviral prescription and hospital length of stay when compared to standard (MPCR) testing in the same influenza season.

## Materials and methods

In 2017, a pilot program was introduced in the New South Wales (NSW) public health system which made available the use of RPCR for influenza (Type A and B) and respiratory syncytial virus (RSV) at all NSW Health Pathology services for the influenza season. The RPCR test was the Cepheid Xpert Xpress Flu/RSV Assay (Cepheid, Sunnyvale, CA) and expected result availability was between one and 4 h. The expected cost per RPCR test was AUD$30 and has in-house verification studies of sensitivity and specificity both at 100%. Standard MPCR testing was carried out using Allplex Respiratory Panel (Seegene Inc., Seoul, South Korea) kit with result availability between one and 4 days. This assay is used to detect Influenza A virus, Influenza B virus, Human respiratory syncytial virus A, Human respiratory syncytial virus B, and subtyping of Influenza A virus (Human Influenza A virus subtype H1, H3, and H1pdm09). The expected cost per MPCR test was AUD$17 and has in-house verification studies of sensitivity and specificity both at 100%. Standard MPCR test was expected to be used where RPCR was not indicated. Memoranda were sent by the virology laboratory to all hospital health services detailing the rationale and laboratory service suggested indications for influenza RPCR testing. The laboratory suggested that RPCR testing be utilised in high risk patients including intensive care unit (ICU) and immunocompromised patients with influenza-like-illness (ILI), emergency department (ED) presentations with respiratory tract infections and inpatients developing symptoms suggestive of influenza or “where rapid laboratory diagnosis of influenza will influence bed management (isolation rooms) and the goals of the Antimicrobial Stewardship Program in containing unnecessary antibiotic use”. Test selection decisions were at the clinician’s discretion.

Retrospective data were collected on an opportunistic cohort of all patients with a positive influenza test result attending Prince of Wales Hospital, Sydney, Australia during the influenza season between 1st June and 30th September 2017. We compared result turnaround time, antibiotic use, antiviral treatment and length of stay (LOS) for both ED only and inpatient admissions between the two assays.

### Data collection

Positive viral PCR results and associated result turnaround time were extracted from the pathology database. Test result turnaround time was calculated from time of sample collection to time of result availability. These results were then matched to corresponding patient electronic hospital medical records for review. Only those with positive RPCR or standard MPCR; greater than 18 years old and who presented to Prince of Wales ED or were admitted to Prince of Wales Hospital were included in this study.

Patient demographic data collected from the electronic medical record included age, sex and degree of co-morbidity which was assessed using Charlson Comorbidity Index [24] based on documented medical history. Patient characteristics considered to be high risk for influenza disease complications were also recorded. These included age greater than 65 years, pregnant, cardiac disease, Down syndrome, obesity (BMI > 30 kg/m^2^), chronic respiratory condition, severe neurological condition, immunocompromise, Aboriginal and Torres Strait Islander, residents of aged care facilities, homeless individuals and any other chronic medical conditions requiring regular medical follow up [25, 26].

Antibiotic prescriptions and duration were extracted from the electronic medical record. Antibiotic indications were based on treating team documentation. Use of antibiotics were deemed inappropriate where a viral infection was confirmed and no clear indication of potential bacterial coinfection recorded including severe influenza disease, clinical deterioration or failure to improve after 3–5 days of antiviral therapy [27]. Medical records were reviewed to assess oseltamivir use. Hospital LOS for both test groups were also collected from the electronic medical record for ED only presentations and those requiring an inpatient hospital admission.

### Data analysis

The main outcomes of interest were antibiotic use, oseltamivir use and hospital LOS in relation to the two available influenza tests. The two groups were also compared on age, Charlson Comorbidity score and mortality rates to ensure they did not differ significantly.

Subgroup analysis was conducted for missed opportunities of oseltamivir use in high risk populations for influenza complications. Those patients with one or more risk factors [25, 26] were identified and assessed as suitable for oseltamivir prescription. These included age greater than 65 years, pregnant, cardiac disease, Down syndrome, obesity (BMI > 30 kg/m^2^), chronic respiratory condition, severe neurological condition, immunocompromise, Aboriginal and Torres Strait Islander, residents of aged care facilities, homeless individuals and any other chronic medical conditions requiring regular medical follow up [25, 26].

Numerical data was assessed using Mann-Whitney test for statistical significance. Categorical data was assessed using Chi-squared (χ^2^) test for statistical significance and odds ratios (OR) with 95% confidence intervals (CI) were calculated. All statistic calculations were completed using VassarStats [28].

Ethics approval was granted by the Human Research Ethics Committee of the South Eastern Sydney Local Health District [HREC ref. no: 2018/ETH00219 (18/161)]. This study methodology was performed in accordance with all local guidelines and regulations.

## Results

During the study period, 484 positive results were identified from the pathology database. Of these, 362 results were positive standard MPCR tests and 122 were positive RPCR tests (Table 1). Patients who had a positive MPCR or RPCR test were not significantly different in terms of age, Charlson Comorbidity score or mortality rate. Turnaround time for results was significantly different between the testing methods with the median time to result availability for the MPCR test significantly longer than the RPCR test (*p* < 0.01).

**Table 1:** Summary of results comparing standard multiplex PCR versus rapid PCR testing for influenza

	Standard Multiplex PCR (***n*** = 362)	Rapid PCR (***n*** = 122)	***P***-value*
**Demographics**			
Age (median years) (IQR**)	74 (51–84)	69 (43–84)	0.28
Gender			0.84
Male (n) (%)	178 (49%)	62 (51%)	
Female (n) (%)	184 (51%)	60 (49%)	
Charlson Comorbidity Index (median score) (IQR)	1 (0–2)	0 (0–2)	0.09
Deaths (n) (%)	6 (2%)	2 (2%)	0.68
**Result availability**			
Test result turnaround time (median hours) (IQR)	22.9 (16.8–38.0)	2.6 (2.0–3.8)	< 0.01
**Discharged from ED (n) (%)**	110 (30%)	33 (27%)	0.56
**Inpatient admission (n) (%)**	252 (70%)	89 (73%)	
**Length of Stay (LOS)**			
Discharged from ED (median days) (IQR)	0.22 (0.15–0.44)	0.25 (0.18–0.31)	0.90
Inpatient admission (median days) (IQR)	5.32 (2.88–11.01)	5.16 (2.72–8.18)	0.72
**Antimicrobial use**			< 0.01
Prescribed (n) (%)	241 (67%)	62 (51%)	
Not prescribed (n) (%)	121 (33%)	60 (49%)	
**Oseltamivir use**			0.02
Prescribed (n) (%)	203 (56%)	84 (69%)	
Not prescribed (n) (%)	159 (44%)	38 (31%)	
**High risk patients (*n*** **= 380)**	*n* = 286	*n* = 94	0.03
Prescribed oseltamivir (n) (%)	180 (63%)	71 (76%)	
Not prescribed oseltamivir (n) (%)	106 (37%)	23 (24%)	

*P-values calculated using χ^2^ test or Mann-Whitney as appropriate. **IQR interquartile range. *ED* Emergency Department

Antibiotic therapy was commenced less frequently in the RPCR than the MPCR group (χ^2^ = 9.01; p < 0.01; OR 0.52; 95% CI 0.34–0.79) and oseltamivir was commenced significantly more frequently in the RPCR group than the MPCR group (χ^2^ = 5.65; *p* = 0.02; OR 1.73; 95% CI 1.12–2.67). More people at risk of complications of influenza were treated with oseltamivir in the RPCR group (76%) than the MPCR group (63%) (χ^2^ = 4.46; *p* = 0.0347; OR 1.81; 95% CI 1.07–3.08).

No significant differences between the two groups were identified in hospital LOS, ED LOS or proportion admitted to hospital.

## Discussion

These data show that the use of RPCR instead of standard MPCR is associated with benefits in antimicrobial stewardship through reduction in unnecessary antibiotic prescribing as well as the provision of oseltamivir therapy for people at high risk of complications with confirmed influenza. Taken together these data show a very positive impact on appropriate antimicrobial prescribing following introduction of a RPCR influenza test at our institution.

Antibiotic prescription significantly decreased from 67 to 51% when RPCR was used indicating a decrease in unnecessary antibiotic prescription. It is well recognised that antimicrobial resistance is a serious global threat to health of which unnecessary antibiotic use is a contributor. A key objective of the World Health Organisation (WHO) Global Action Plan on Antimicrobial Resistance is to optimise the use of antimicrobial medications recommending effective and rapid diagnostic tools as part of this plan [29]. In line with WHO guidelines, our results would support the use of RPCR as opposed to standard MPCR as an effective diagnostic tool to improve antimicrobial stewardship. Rapid testing has previously been suggested as a means to improve antibiotic utilisation however, previous studies have been performed using a rapid antigen test with a lower sensitivity than either of the PCR-based tests used in this study and were performed in outpatient ED, paediatric or resource limited settings [11, 30–33]. Our findings extend on and are supported by a small number of previous studies that suggested a reduction in antibiotic usage with RPCR testing [14, 17–19, 23]. In comparison to our study these were are mostly small studies assessing effects on ED metrics and included non-comparative cohort studies [14–16] or used historical controls [17–23]. In particular, one trial found antimicrobial stewardship improvement only occurred in their paediatric and not in their adult population [19]. Notably there has been one large, UK hospital-based, open-label randomised controlled trial comparing outcomes of routine RPCR at presentation of respiratory illness versus standard clinical care. In this UK study, those allocated to the standard clinical care group were provided treatment at the discretion of treating teams including potential conventional respiratory viral PCR testing as well as antimicrobial use. This study found that routine RPCR did not reduce duration of antibiotics overall, but did find that patients in the RPCR group received either single dose or shorter courses of antibiotics compared to the control group [34]. Though they found higher proportions of single dose/shorter courses of antibiotics in their RPCR group, the effect appeared to have been muted in the overall duration of antibiotics due to clinical populations in their study, such as pneumonia, requiring consistently longer courses of antibiotics. Our findings would suggest that earlier diagnosis could alter clinical management such that unnecessary antibiotic use can be decreased. In reducing unnecessary antibiotics, not only will this reduce antimicrobial resistance, but patients and hospitals could thereby reduce known risks associated with antibiotic overuse such as antibiotic associated adverse effects, re-attendance due to infectious disease, increased healthcare costs or increased length of stay [8].

Subgroup analyses of higher risk patients for complications of influenza would suggest that RPCR testing lead to fewer missed opportunities for oseltamivir treatment compared to MPCR testing (24% vs 37%, respectively). Early oseltamivir use has benefits in improving resolution of clinical symptoms, reducing risk of lower respiratory tract infections and prevention of hospital admission when used in influenza positive patients [35]. Current Australian Therapeutic Guidelines recommend considering oseltamivir treatment for individuals at risk of poor outcomes such as elderly, pregnant women, immunocompromised, etc. [2] A recent randomised controlled trial demonstrated oseltamivir treatment in the older, co-morbid population can improve recovery by two to 3 days, further supporting the need to target early therapy especially in these higher risk individuals [6]. This is the only study to our knowledge that has looked at the use of RPCR diagnostic testing to optimise oseltamivir use in high risk individuals for complications of disease. A small, single centre US study comparing MPCR to RPCR, found that negative RPCR results lead to reduced duration of empiric antiviral therapy with no difference in intensive care admissions or antibiotic use [22]. This study, however, examined both positive and negative influenza results and those that were positive only accounted for 11% of the study population [22]. Small non-comparative studies, within the ED setting alone, have also suggested that utilisation of RPCR may improve oseltamivir prescription [15, 16, 21, 36]. Both our study, in conjunction with the US study and non-comparative studies would support the idea that a RPCR test compared to a MPCR test improves the utilisation of oseltamivir.

Utilisation of either diagnostic test did not appear to affect LOS in the ED or as an inpatient, as neither result achieved statistical significance. Logistical factors occurring within the ED or inpatient wards that were not assessed within this study may have played a role. There have been a small number of studies published on RPCR testing and its impact on LOS both in ED and inpatient settings [34, 37–39]. Overall, these studies have been of varying design and have demonstrated different outcomes. Factors identified that affected LOS included being provided positive RPCR results during hospital stay or delays in timing of patient diagnostic sampling [38, 39]. Our study did not examine time difference of patient presentation to sampling nor had we analysed patient disposition at time of result availability which may have impacted LOS and contributed to our findings. The present study had a smaller sample size and may not have sufficient power to detect a difference in LOS.

This study’s limitations include that data were from a single hospital, were collected retrospectively and could have been subject to selection bias at the time of test selection (MPCR vs RPCR). However, to try and account for this we checked to ensure that the groups did not differ significantly on the basis of age, mortality or morbidity. Data were collected through reviewing each individual’s medical record, and therefore relied on the accuracy of documentation at time of presentation. Medical history and clinical data were extracted from the medical records, therefore, to minimise bias, the same author collected all the data reducing variability in interpretation. Population numbers in the RPCR group were lower than the multiplex group, likely reflecting lower utilisation after introduction of a new testing method.

Future studies could utilise larger, prospective, randomised trials with direct comparison of standard multiplex and rapid PCR testing to monitor outcomes. Larger uptake in test utilisation and influenza positive cases may improve the statistical power and would be of interest in comparison to this study. Research questions that could be further explored in a prospective randomised trial include effects on bed management, patient flow and an exploration of the multi-level factors that impact LOS. Cost-benefit analyses was not assessed in this study. We note that the RPCR is significantly more expensive than the standard MPCR with an expected cost of approximately AUD$30 versus AUD$17, respectively. Future studies could examine the cost-benefit of the RPCR test.

## Conclusion

The use of a rapid influenza PCR test was associated with reduced inappropriate antibiotic use and increased appropriate oseltamivir use in patients at high risk of influenza complications. A cost benefit analysis and review of the RPCR in a typical influenza season could examine better the impact on length of stay and bed management.

## Data Availability

The datasets used and/or analysed during the current study are available from the corresponding author on reasonable request.
